# Bidirectional Automated Texting for Cardiovascular Health Among People Living With HIV: Observational Cohort Analysis of a Stepped-Wedge Cluster Randomized Controlled Trial

**DOI:** 10.2196/69098

**Published:** 2026-07-14

**Authors:** Mechelle Sanders, Donald Harrington, Emma Sass, Marie Thomas, Tameir Holder, Yiqi Tian, Brent Johnson, Andrea Cassells, Jonathan N Tobin, Kevin Fiscella

**Affiliations:** 1 Department of Family Medicine, University of Rochester Medical Center, Rochester, NY, United States, 1 585-324-4566, 1 585-473-2245; 2 Department of Biostatistics and Computational Biology, University of Rochester Medical Center, Rochester, United States; 3 Clinical Directors Network, New York, NY, United States; 4 Department of Biostatistics, University of Rochester Medical Center, Rochester, NY, United States; 5 The Rockefeller University, The Rockefeller University Center for Clinical and Translational Science, New York, NY, United States

**Keywords:** mobile health, mHealth, digital intervention, text messaging, HIV, cardiovascular disease

## Abstract

**Background:**

Feasible and potentially scalable strategies are needed to address the growing cardiovascular disease (CVD) risk among people living with HIV. Bidirectional automated texting (BAT) programs that remind and encourage adherence to evidence-based CVD-reducing interventions represent a potentially scalable strategy, but data on their feasibility are lacking.

**Objective:**

The goal of the study was to determine whether participant sociodemographic factors and technological constraints influenced engagement with a BAT CVD prevention program by people living with HIV. We conducted an observational cohort analysis embedded within a stepped-wedge cluster randomized trial.

**Methods:**

The BAT program was designed to address the “Million Hearts” ABCS (aspirin therapy, blood pressure control, cholesterol management, and smoking cessation) of cardiovascular health. The parent study was a stepped-wedge randomized trial that rolled out in 3 wedges across 8 practice sites that provided care to people living with HIV. Participants received and could engage with the text messages weekly using their own phones during the study period. We used a zero-inflated negative binomial model to identify factors associated with participants sending text messages during the study.

**Results:**

Of the 471 participants, 94% owned a smartphone capable of text messaging, and 70% reported monthly incomes less than US $1500. Overall, 60.3% (n=284) engaged with the BAT program at least once. Regarding texting behavior, participants aged ≥65 years were more likely to send a text than those aged <50 years (*P*=.047), although age did not influence the number of texts sent. White participants showed lower texting intensity than Black participants (incidence rate ratio 0.69; *P*=.04).

**Conclusions:**

Overall, 60.3% (n=284) of the participants in the study engaged with BAT at least once. The BAT intervention for ABCS appears to be a feasible intervention for people living with HIV. Only a few factors were associated with sending a text or with the number of text messages sent.

## Introduction

Use of highly effective antiretroviral therapy has dramatically reduced deaths from AIDS-related causes, yielding an aging population among people living with HIV [1,2]. Cardiovascular disease (CVD) risk is up to 2 times greater among people living with HIV compared with the general population [3,4]. Bidirectional automated texting (BAT) has been successfully used for multiple types of health interventions, including tobacco cessation, blood pressure control, cancer screening, medication adherence, and HIV treatment [5-10]. BAT allows users to respond to confirm medical appointments, answer questions, report symptoms, and share health updates in real time or at their convenience, enabling efficient and ongoing asynchronous communication between patients and their health care team [5-7]. BAT can also be used to collect health information through text messages. It is a potentially scalable tool for communicating with patients outside the clinical encounter while enabling the provision of tailored information based on responses to prior text messages [8].

A systematic review found that text-messaging programs for CVD can help people better manage their heart disease risk factors [9]. Given this, the growing risk of CVD among people living with HIV may be substantively reduced by using evidence-based BAT programs that remind and encourage adherence to evidence-based CVD-reducing interventions [10]. Compared with other types of engagement, texting offers potentially higher reach relative to cost and is scalable due to its low marginal costs once established [10-13]. Beyond the general efficacy, specific trials have also explored the practical utility and user acceptance of texting interventions for preventing and managing CVD [14,15]. For example, in one study, participants received 1 text per week about general CVD knowledge, blood pressure control, medication adherence, physical activity, healthy diet, and smoking cessation (only for smokers). The findings highlighted strong user satisfaction, with nearly all participants reporting that the text messages were useful (96.1%), easy to understand (98.8%), and appropriate in frequency (93.8%). Participants also reported a high willingness to receive future messages (94.8%) [16].

Several studies have shown that text messaging can improve medication adherence and appointment attendance [17-21]. However, evidence for its impact on sexually transmitted infection and HIV prevention and treatment remains mixed [22,23]. McLaughlin et al [24] proposed a framework for creating educational text messages around primary prevention of CVD in people living with HIV, but randomized trials are still needed to confirm its effectiveness. To address this gap, we analyzed feasibility data from an observational cohort nested within a stepped-wedge trial to identify factors influencing engagement among people living with HIV with CVD prevention text messages.

## Methods

### Population

The HIV ABCS (aspirin therapy, blood pressure control, cholesterol management, and smoking cessation) trial, which was part of PRECluDE (Implementation Research to Develop Interventions for People Living With HIV), a consortium of National Heart, Lung, and Blood Institute funded trials, has been previously described (ClinicalTrials.gov NCT03902431) [25,26]. Participants were recruited from 8 geographically diverse sites across 3 locations: 4 in New York City, New York; 2 in Rochester, New York; and 2 in Dallas, Texas. These sites included academic medical centers, freestanding HIV practices, and Federally Qualified Health Centers (FQHCs). Participants were eligible if they had English or Spanish language proficiency sufficient to read text messages and a personal mobile phone. We enrolled 471 participants aged 40 to 75 years (by July 1, 2018) with a 10-year atherosclerotic CVD (ASCVD) risk of at least 5% without known cardiovascular or cerebrovascular disease (eg, stroke, myocardial infarction, and coronary artery disease), based on eligibility criteria from the ASCVD risk calculator [27].

### Intervention

This study was an observational cohort nested within the parent stepped-wedge trial. The parent stepped-wedge trial was conducted between 2019 and 2023. This design used a multicomponent strategy to promote the uptake of the US Department of Health and Human Services’ “Million Hearts” ABCS campaign for cardiovascular health [28]. This design involved 8 sites divided into 3 wedges, with each wedge separated by 6 to 8 months.

**Figure 1.**
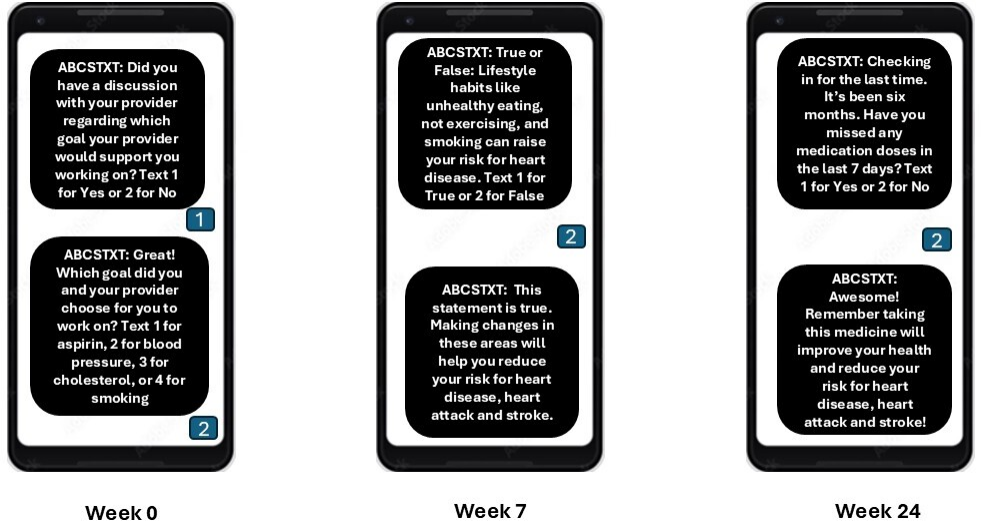
Examples of goal-setting text messages sent to the participants in the HIV ABCS (aspirin therapy, blood pressure control, cholesterol management, and smoking cessation) and their responses at different time points.

For the patient-facing component, BAT messages were delivered by a commercial vendor (Mosio). To develop the BAT for CVD prevention for people living with HIV, we generated a library of evidence-based text messages for each of the ABCS. The messages included potential behavior change techniques, knowledge about the ABCS, personal affirmation, self-efficacy, adherence support, and the importance of goal commitment (Figure 1). Mosio sent messages in either English or Spanish, depending on participants’ language preferences.

Participants engaged with these text messages for a maximum of 6 months, regardless of their assigned wedge. Message frequency was initially set at 1 to 3 texts per day for the first 4 months, then tapered to 1 to 2 texts per week. This frequency was also adaptive, with additional texts sent based on participant responses to prompts, such as indicating a need for more information or incorrect answers to CVD knowledge checks. This was embedded in the program’s branching logic. To encourage sustained engagement throughout the 6-month texting period, participants were eligible for 3 US $5 incentives (up to US $15 overall) if they responded to specific text messages that were randomly selected from the automated message library [29].

### Demographic and Psychosocial Variables

Research assistants collected data from participants on sex at birth, race, ethnicity, preferred language, and income. Participants also reported their chronic conditions (hypertension or diabetes) and completed validated scales: the Patient Activation Measure (PAM), the Confusion, Hubbub, and Order Scale (CHAOS), and the Subjective Numeracy Scale (SNS) [30-32]. The PAM assessed patients’ knowledge, skills, and confidence in managing their health. The CHAOS scale evaluated household environment disorganization and its potential impact on texting behaviors, while the SNS measured patients’ abilities to understand and use health-related information effectively. Together, these instruments provided a comprehensive assessment of factors influencing self-management and texting engagement. Both the PAM and SNS have been evaluated in similar populations [33,34].

### Smartphone and Text Message Use and Skills

We developed 8 questions to evaluate participants’ smartphone use and skills, covering ownership, length of use, acquisition type, self-rated skill level, frequency of service interruption, ability to text during interruptions, and time since the last interruption. For instance, participants reported years of smartphone ownership or use, with options ranging from “0‐3 years” to “more than 7 years.” Participants also rated their smartphone skill from “Beginner” (limited to calls and texts) to “Expert” (full proficiency, including settings). Phone acquisition options included “Purchase,” “Gift,” or “Government Plan.” Participants indicated how they paid for their data service plan (eg, postpaid, prepaid, limited minutes, or other options). The frequency of phone or data service interruptions was assessed with options ranging from “every month by the first week” to “never.” Finally, the survey asked about unlimited texting capability and the ability to receive texts during service interruptions, with “Yes,” “No,” and “Unsure” as response options.

### Texting Engagement

Previous studies of text-messaging interventions have shown that outcomes often follow a dose-response pattern, where greater engagement can lead to larger improvements in the variables of interest [35-37]. To assess patient engagement with BAT, we counted the number of text messages patients sent. We created two engagement measures: (1) a binary indicator of any engagement (sent at least 1 message vs no messages) and (2) a continuous count of messages among participants who sent at least 1 message.

### ABCS Goal Choice

We identified the participants’ ABCS goal based on their report within the BAT system. Participants specifically reported the goal that they had discussed and mutually agreed upon with their clinician.

### Data Analysis

We performed all analyses using R (version 4.4.2; R Foundation for Statistical Computing). All statistical tests were 2-tailed and based on a 5% significance level. We calculated descriptive statistics and presented these as means and SDs where appropriate.

We used a zero-inflated negative binomial (ZINB) model to assess factors associated with participants sending a text. Our model accounted for excess zeros (eg, never sending a text) and overdispersion. Covariates for the model were selected using a 2-step process: first, variables were screened for predictive importance using Bayesian additive regression trees (BART) with the threshold of 0.4; second, the final model included variables identified by BART as well as standard sociodemographic adjusters (eg, age, race, and sex) important to the study design and supported by existing literature. Ethnicity was screened out by the BART procedure and was not retained in the final model owing to its collinearity with race. The count model examined the frequency of texts sent among those who sent at least 1 text (incidence rate ratio), while the inflation model predicted the likelihood of never sending a text versus sending any texts (odds ratio).

### Ethical Considerations

The study was approved by the institutional review board at the University of Rochester Medical Center (approval number STUDY00003679), with reliance review at each of the participating sites. Informed consent was obtained from each participant. Participants could receive up to US $80 for this study: US $40 for the baseline visit, US $10 for the second visit, US $15 for the third visit, and up to an additional US $15 (3 US $5 cards) for responding to randomly incentivized text messages, which were provided at the second or third visit.

## Results

### Participants

The participants were middle-aged, that is, 55 years or older (336/470, 71.5%), male (321/471, 68.2%), and Black (273/470, 58.1%). Among participants with available income data, 48.7% (211/433) reported a monthly household income of less than US $1000 per month (Table 1).

**Table 1.** Baseline participant characteristics^a^.

Characteristics	Participants
Demographics	
Age (y; n=470), n (%)	
40‐49	50 (10.6)
50‐54	84 (17.9)
55‐59	134 (28.5)
60‐64	114 (24.3)
≥65	88 (18.7)
Education (n=443), n (%)	
Less than high school	107 (24.2)
High school graduate	141 (31.8)
Higher than high school	195 (44)
Monthly household income (US $; n=433), n (%)	
<1000	211 (48.7)
1000‐1499	89 (20.6)
1500‐1999	55 (12.7)
>2000	78 (18)
Sex at birth (n=471), n (%)	
Male	321 (68.2)
Female	149 (31.6)
Decline to state	1 (0.2)
Race (n=470), n (%)	
Black or African American	273 (58.1)
White	136 (28.9)
Other	61 (13)
Ethnicity (Hispanic or Latino; n=434), n (%)	87 (20)
Clinical characteristics	
Blood pressure at baseline (mm Hg), mean (SD)	
Systolic (n=470)	135.32 (18.2)
Diastolic (n=464)	82.52 (10.7)
Treated for hypertension (n=470), n (%)	293 (62.3)
Has diabetes (n=470), n (%)	154 (32.8)
Smoking status (n=470), n (%)	
Current	195 (41.5)
Never	147 (31.3)
Former	128 (27.2)
Regular aspirin use (n=465), n (%)	104 (22.4)
Treated with statin (n=466), n (%)	266 (57.1)
Total cholesterol (mg/dL; n=470), mean (SD)	186.2 (37.5)
Survey measures, mean (SD)	
Health literacy score (n=412)	4.48 (1.3)
Subjective Numeracy Scale score (1-6; n=446)	3.94 (1.0)
Confusion, Hubbub, and Order Scale score (0‐30; n=433)	13.98 (4.3)
Patient Activation Measure raw score (13-52; n=447)	43.55 (5.9)
Technology access and skills	
Own or have access to a smartphone (n=446), n (%)	417 (93.5)
Duration of owning or using a smartphone (y; n=439), n (%)	
0‐3	141 (32.1)
4‐7	103 (23.5)
>7	195 (44.4)
Smartphone acquisition (n=446), n (%)	
Purchased	359 (80.5)
Gift	72 (16.1)
Government plan	15 (3.4)
Self-rated smartphone skill (n=439), n (%)	
Beginner (calls and text only)	117 (26.7)
Advanced beginner (calls and text and internet search)	60 (13.7)
Intermediate (calls and texts, internet search, and downloading apps)	110 (25.1)
Advanced (calls and texts, internet search, downloading apps, and changing settings and passwords)	75 (17.1)
Expert (all of the skills and more)	77 (17.6)
Phone or data service interruption frequency (n=444), n (%)	
Every month, first week	34 (7.8)
Every month, second week	8 (1.8)
Every month, third week	11 (2.5)
Rarely	87 (19.6)
Never	304 (68.5)
Unlimited texting capability (n=446), n (%)	383 (85.9)
Able to receive texts when service interrupted (n=441), n (%)	211 (47.8)
Service interrupted in past 4 months (n=314), n (%)	31 (9.9)
ABCS^b^ goal choice (n=448), n (%)	
Aspirin	28 (6.3)
Blood pressure	136 (30.4)
Cholesterol	169 (37.8)
Smoking	115 (25.8)
Primary outcome measure	
Did the participant send a text message? (n=471), n (%)	284 (60.3)
Message count (among those that sent a text; n=284), mean (SD)	29.26 (34.2)
Participants per site (n=471), n (%)	
Site 1	109 (23.1)
Site 2	43 (9.1)
Site 3	57 (12.1)
Site 4	40 (8.5)
Site 5	31 (6.7)
Site 6	42 (8.9)
Site 7	110 (23.4)
Site 8	39 (8.3)

a The sample sizes vary across variables because not all participants provided responses for every measure, including missing data in electronic health records.

b ABCS: aspirin therapy, blood pressure control, cholesterol management, and smoking cessation.

A total of 93.5% (417/446) of participants owned smartphones and 32.1% (141/439) had owned or used one for ≤3 years. Most participants (359/446, 80.5%) purchased their phone, whereas 3.4% (15/446) received it through a government plan. Most participants (304/444, 68.5%) never had service interruptions. Self-rated smartphone skills varied, with 26.7% (117/439) reporting being beginners and 34.6% (152/439) as advanced or expert (Table 1).

### ZINB Model

Out of a total of 39 potential variables, BART selected 5 (12.8%) variables as important predictors of texting: smoking status, clinical practice site, self-rated smartphone skill, length of smartphone use, and final goal (ABCS), but none of the 4 survey measures. Together with age, sex, and race, the final model included 8 independent predictors. We fit the ZINB model to the text count outcome and the above 8 key covariates, after excluding 38 participants due to missing data (Table 2).

**Table 2.** Zero-Inflation model: predictors of texting engagement^a^.

Predictor	Never texted, OR^b^ (95% CI)	*P* value	Text count, IRR^c^ (95% CI)	*P* value
Age (y)				
<50	Reference	—^d^	—	—
50‐54	0.63 (0.24-1.67)	.36	1.13 (0.61-2.09)	.71
55‐59	0.56 (0.23-1.40)	.21	1.28 (0.72-2.27)	.44
60‐64	0.78 (0.30-2.00)	.61	0.87 (0.48-1.56)	.64
≥65	0.33 (0.11-0.99)	.047	0.80 (0.44-1.47)	.48
Sex				
Female	Reference	—	—	—
Male	0.78 (0.42-1.46)	.44	0.81 (0.58-1.12)	.22
Race				
Black or African American	Reference	—	—	—
White	0.65 (0.30-1.44)	.29	0.69 (0.49-0.98)	.04
Other	1.14 (0.51-2.54)	.74	0.84 (0.54-1.31)	.44
Smoking status				
Current	Reference	—	—	—
Former	1.33 (0.59-2.99)	.49	1.18 (0.77-1.79)	.45
Never	1.32 (0.61-2.82)	.48	1.02 (0.67-1.51)	.92
Study site				
Site 1	Reference	—	—	—
Site 2	0.91 (0.38-2.19)	.84	0.55 (0.28-1.08)	.08
Site 3	0.22 (0.08-0.59)	.003	0.34 (0.18-0.62)	<.001
Site 4	0.14 (0.04-0.46)	.001	0.36 (0.20-0.63)	<.001
Site 5	0.54 (0.20-1.48)	.23	0.67 (0.32-1.39)	.28
Site 6	0.20 (0.07-0.59)	.004	0.76 (0.42-1.37)	.36
Site 7	0.08 (0.03-0.21)	<.001	1.23 (0.81-1.89)	.33
Site 8	0.09 (0.02-0.40)	.002	0.59 (0.34-1.02)	.06
Self-rated smartphone skill				
Beginner	Reference	—	—	—
Advanced beginner	0.76 (0.33-1.79)	.53	1.19 (0.76-1.84)	.45
Intermediate	0.70 (0.33-1.47)	.35	1.33 (0.88-2.00)	.18
Advanced	0.74 (0.30-1.83)	.52	1.19 (0.75-1.89)	.46
Expert	0.39 (0.14-1.09)	.07	1.20 (0.77-1.85)	.42
Length of smartphone use (y)				
<4	Reference	—	—	—
4‐7	0.91 (0.45-1.83)	.79	1.27 (0.87-1.86)	.22
>7	0.72 (0.36-1.43)	.34	1.04 (0.74-1.46)	.81
ABCS^e^ goal				
Cholesterol	Reference	—	—	—
Aspirin	0.25 (0.07-0.83)	.02	1.45 (0.83-2.51)	.19
Blood pressure	0.86 (0.46-1.61)	.63	0.90 (0.65-1.25)	.53
Smoking	1.39 (0.59-3.25)	.45	1.06 (0.68-1.64)	.80

a The point estimate for the natural logarithm of the size parameter in the statistical model was −0.03 (95% CI −0.26 to 0.19; *P*=.77).

b OR: odds ratio.

c IRR: incidence rate ratio.

d Reference category (zero inflation).

e ABCS: aspirin therapy, blood pressure control, cholesterol management, and smoking cessation.

The odds of ever texting were 3-fold higher for participants aged ≥65 years than those aged <50 years (*P*=.047). However, age was not associated with the number of texts sent among participants who sent at least 1 text. Regarding race, we observed no statistically significant impact on whether a participant ever sent a text. However, among those who texted, White participants sent approximately 31% fewer text messages than Black participants (*P*=.04). Participants at 5 sites were more likely to send at least 1 text compared with those at the reference site. However, among participants who texted at least once, those from two of these same sites sent fewer text messages.

## Discussion

### Principal Results

This study explored factors affecting people living with HIV engagement with a BAT CVD prevention texting program among people living with HIV. In our ZINB model, older age, HIV care site, and selecting aspirin as a goal were associated with texting participation. The association between site and texting engagement warrants further investigation. It is not clear why, after controlling for patient-level factors, participants at 5 sites were more likely to send at least 1 text, and why those at 2 of these sites who texted at least once sent *fewer* text messages. This unexpected finding may serve as a surrogate for unmeasured site-specific factors such as differences in staffing, changes in on-site technical support for texting, prior digital health experience, or the quality of study implementation. These unmeasured factors may have been stronger predictors of engagement than patient-level demographic characteristics. The association with aspirin may reflect the complexity of the 2016 US Preventive Services Task Force’s aspirin recommendations: “The USPSTF recommends initiating low-dose aspirin use for the primary prevention of cardiovascular disease (CVD) and colorectal cancer in adults aged 50 to 59 years who have a 10% or greater 10-year CVD risk, are not at increased risk for bleeding, have a life expectancy of at least 10 years, and are willing to take low-dose aspirin daily for at least 10 years [38].” Participants selecting aspirin may have texted more due to this added complexity. While our data cannot definitively assess these explanations, future research incorporating qualitative methods could investigate these contextual factors more deeply.

### Comparison With Prior Work

Our results align with previous studies demonstrating BAT is feasible and readily used among historically lower digitally literate participants, that is, those who are older, have low income, and have less education [39]. Our results also reflect the marked increase in smartphone ownership across all demographic groups, consistent with broader trends reported in the literature [40]. Our analysis included primarily participants who did not report monthly interruptions in mobile phone service. This approach allowed us to examine patient engagement within a cohort largely free from connectivity challenges, which previous studies have identified as a significant barrier [41,42]. Building on prior work by our group and others that indicates the feasibility of providing digital literacy training to low-income people living with HIV [43,44], our findings further support the potential for technology-assisted interventions to increase engagement with care. This suggests a promising avenue for future intervention development, potentially increasing engagement with care, and facilitating the evaluation of both effectiveness and implementation outcomes.

While race did not significantly influence the likelihood of a participant sending any text, we observed that among those who did text, White participants sent fewer text messages than Black participants. This finding is consistent with earlier data from 2010 showing that Black and Hispanic individuals who use text messaging text more on average than White individuals, with a median of 10 text messages per day for Black and Hispanic individuals compared with 5 text messages per day for White individuals [10]. To mitigate language barriers a priori, our research staff who enrolled Spanish-speaking patients were bilingual, and BAT messages were translated and back-translated into Spanish. However, our subsample of Spanish-speaking patients was modest, and we cannot rule out the need for further cultural or linguistic adaptations to the text messages.

### Limitations and Strengths

One limitation of this study is the lack of qualitative data at the patient, clinician, and site levels. We were limited to quantitative information and may have missed important contextual factors that could help explain the differences observed across sites. For example, at the start of the intervention, participants were able to self-select the best time of day to receive texts. However, participants’ preferred texting times may have changed over time (eg, due to changes in employment hours), potentially limiting their ability to engage with the intervention. Additionally, it is possible that participants saw the texts, read them, and decided not to engage with the text. Lack of engagement does not necessarily preclude them from following through with their ABCS goals. In univariate analyses, we observed that participants experiencing service interruptions were less likely to send messages (*P*<.001). However, neither service interruption variable was important in the BART variable selection procedure after adjusting for the other key variables. Importantly, although we adopted a complete-case analysis in the ZINB model, the BART procedure performs feature selection without this assumption under a missing at random assumption and, therefore, did not remove records with missing values when selecting important features. However, the BART method for handling missing data is biased toward the null, which implies that BART would tend to shrink a nonnegligible correlation closer to zero in the presence of missing data [45]. Consequently, our measure of engagement may underestimate true engagement among those facing connectivity challenges. The prevalence of service interruptions itself may be underestimated if those most affected were also less likely to report them. The study used modest random monetary incentives to increase texting engagement, with participants able to receive an additional US $15 for responding to randomly incentivized messages during the 6-month intervention. This approach may have influenced their motivation to text, and it is unclear whether this motivation would persist in a real-world setting without such incentives. Moreover, we were unable to control for these random incentives in our analysis, making them a potential confounder for the number of texts sent. A separate limitation is that we were unable to control for the adaptive logic of the text messaging (eg, additional prompts based on responses), which may have biased the estimated associations with texting frequency or engagement.

Despite these limitations, the strengths of our study include the availability of both Spanish and English versions of the text messages, a geographically diverse participant population, multiple types of care settings (eg, FQHCs and academic health centers), and a large sample size that enhances the generalizability of the findings.

### Conclusions

A BAT intervention for the ABCS of heart health appears to be a promising tool for engaging people living with HIV. Overall, 60.3% (284/471) of the participants in the study were able to engage with the texting platform at least once. Surprisingly, few factors were associated with sending a text or with the number of text messages sent.

BAT is feasible to implement among low-income people living with HIV. Future studies should examine contextual factors that may have influenced engagement but were not captured in our research, particularly the role of personal assistance. Given the increasing reliance on text messaging by health systems for patient communication (eg, scheduling, reminders, and results), future efforts should explore how BAT interventions can be integrated into clinical workflows to reinforce CVD prevention, particularly among patients with low incomes.

## Abbreviations

ABCS: aspirin therapy, blood pressure control, cholesterol management, and smoking cessation
ASCVD: atherosclerotic cardiovascular disease
BART: Bayesian additive regression trees
BAT: bidirectional automated texting
CHAOS: Confusion, Hubbub, and Order Scale
CVD: cardiovascular disease
FQHC: Federally Qualified Health Center
PAM: Patient Activation Measure
SNS: Subjective Numeracy Scale
ZINB: zero-inflated negative binomial
